# Waterlogging Tolerance of *Actinidia valvata Dunn* Is Associated with High Activities of Pyruvate Decarboxylase, Alcohol Dehydrogenase and Antioxidant Enzymes

**DOI:** 10.3390/plants12152872

**Published:** 2023-08-04

**Authors:** Minxia Gao, Chaoyue Gai, Xinyu Li, Xin Feng, Ruilian Lai, Yuanyuan Song, Rensen Zeng, Daoqian Chen, Yiting Chen

**Affiliations:** 1 Fruit Research Institute, Fujian Academy of Agricultural Sciences, Fuzhou 350013, China; 2 Key Laboratory of Ministry of Education for Genetics, Breeding and Multiple Utilization of Crops, College of Agriculture, Fujian Agriculture and Forestry University, Jinshan, Fuzhou 350002, China

**Keywords:** kiwifruit, waterlogging, aerenchyma, anaerobic fermentation, pyruvate decarboxylase, alcohol dehydrogenase, antioxidant enzyme, antioxidant ability

## Abstract

Kiwifruit (*Actinidia* spp.) is susceptible to waterlogging stress. Although abundant wild germplasm resources exist among *Actinidia* plants for improving the waterlogging tolerance of kiwifruit cultivars, the underlying mechanisms remain largely unknown. Here, a comparative study was undertaken using one wild germplasm, Maorenshen (*A. valvata* Dunn, MRS), and one cultivar, Miliang-1 (*A. chinensis* var. *deliciosa* (A.Chev.) A.Chev. cv. Miliang-1, ML). Under stress, the ML plantlets were seriously damaged with wilted chlorotic leaves and blackened rotten roots, whereas the symptoms of injury in the MRS plantlets were much fewer, along with higher photosynthetic rates, chlorophyll fluorescence characteristics and root activity under stress conditions. However, neither aerenchyma in the root nor adventitious roots appeared in both germplasms upon stress exposure. The activities of pyruvate decarboxylase (PDC) and alcohol dehydrogenase (ADH), as well as their transcript levels, were constitutively higher in MRS than those in ML under both normal and stress conditions. Waterlogging stress significantly enhanced the PDC and ADH enzyme activities in both germplasms, which were 60.8% and 22.4% higher in the MRS roots than those in the ML roots under waterlogging stress, respectively. Moreover, MRS displayed higher activities of antioxidant enzymes, including SOD, CAT, and APX, as well as DPPH-radical scavenging ability, and decreased H_2_O_2_ and MDA accumulation under both normal and stress conditions. Our findings suggest that the waterlogging tolerance of the wild *A. valvata* germplasm was associated with high PDC and ADH, as well as antioxidant ability.

## 1. Introduction

Kiwifruit is one of the most popular fruits worldwide due to its delicious taste and rich nutrient content, as well as its high vitamin C content. However, the majority of current kiwifruit cultivars (*Actinidia* spp.) are susceptible to waterlogging stress [1,2,3]. Kiwifruit plants generally suffer from excess rainfall during the rainy season in their main planting areas, especially in Southeast China [4,5]. Waterlogging negatively impacts on the fruit yield and even threatens the survival of trees by reducing vigor and increasing their susceptibility to disease [3,6,7]. It is predicted that both the severity and frequency of waterlogging will rise worldwide as a result of global warming. Therefore, there is an urgent need to breed kiwifruit cultivars with greater efficiency and effective waterlogging adaptations. To achieve this goal, it is necessary to understand the waterlogging response and adaptation mechanisms of plants under stress.

Waterlogging stress results in root oxygen deprivation, which threatens the growth and survival of plants. Waterlogging stress generally alters a series of plant physiological traits, such as photosynthesis, water status, nutrient uptake, reactive oxygen homeostasis and subsequently inhibits plant growth and development [8,9,10]. Plants have developed metabolic and/or morphological mechanisms to cope with low oxygen conditions [6,10,11]. Under waterlogging stress, the induction of adventitious roots and aerenchyma contribute to the transport of oxygen to the insufficiently oxygenated zones [10,11,12]. The plant energy metabolism is shifted from an aerobic to an anaerobic state to supply ATP under oxygen deprivation conditions [10,11]. Omics analyses have shown that transcripts of the genes related to the glycolytic and anaerobic fermentation pathways, including alcohol dehydrogenase (ADH) and pyruvate decarboxylase (PDC)-encoding genes, are significantly enriched in response to hypoxia [1,13,14]. The soybean seed germination ability was enhanced by the overexpression of *GmADH2* under waterlogging [15]. The overexpression of any one of kiwifruit *AdADH1*, *AdADH2*, *AdPDC1* or *AdPDC2* from *A. deliciosa* (*A. chinensis* var. *deliciosa* (A.Chev.) A.Chev.) could enhance the waterlogging tolerance in *Arabidopsis* [5,16,17]. Recently, it was reported that the overexpression of *AdRAP2.3* gene, an ethylene response factor VII from *A. deliciosa*, conferred waterlogging stress tolerance through improving the expression levels of the *PDC* and *ADH* genes [18].

A rich genetic variability and abundant wild germplasm resources exist among *Actinidia* plants [19,20,21]. However, the waterlogging adaptation strategy of these germplasm resources remains largely unknown. An increased understanding of the mechanisms involved in waterlogging tolerance in wild germplasms is conducive to developing kiwifruit cultivars or rootstocks tolerant to waterlogging. The wild germplasm Maorenshen (*A. valvata* Dunn, MRS) and the cultivar Miliang-1 (*A. deliciosa* cv. Miliang-1, ML) contrasted markedly with one another with respect to their waterlogging tolerance in our preliminary experiments. The former appears to be able to survive in episodes of waterlogging, whereas the latter is a traditional cultivar with high productivity but is susceptible to waterlogging. Here, a comparative study on the morphological, anatomical, physiological and molecular responses to waterlogging of the two contrasting kiwifruit germplasms was conducted. The aim of this study was to test our hypothesis that the waterlogging tolerance of the wild germplasm from *A. valvata* is associated with metabolic adaptions with enhanced ethanolic fermentation and antioxidant abilities during waterlogging.

## 2. Results

### 2.1. Morphological Responses to Waterlogging

**Figure 1:**
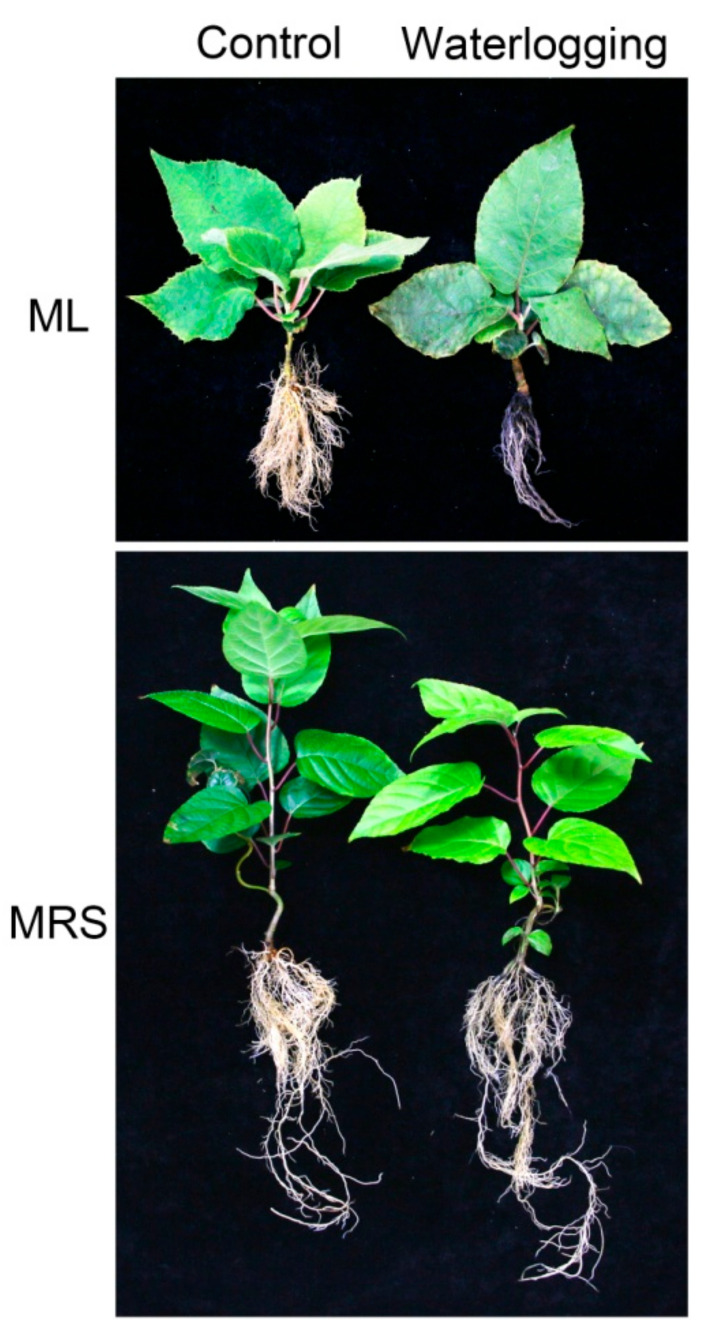
Phenotypes of MRS and ML plantlets subjected to waterlogging stress for 3 weeks. MRS: Maorenshen, a wild germplasm from *Actinidia valvata* Dunn. ML: Miliang-1, a cultivar from *Actinidia chinensis* var. *deliciosa* (A.Chev.) A.Chev.

Under normal conditions, the two germplasms showed significant differences in the leaf, root and stem morphologies. Compared with ML, the MRS plantlets had hairless leaves, deeper roots and more elongated stems. After 3 weeks of waterlogging stress, the ML plantlets were severely damaged with wilted chlorotic leaves and blackened rotten roots. The symptoms of injury in the MRS plantlets were fewer. No wilted leaves or rotten roots were observed in the MRS plantlets (Figure 1). After 3 weeks of waterlogging stress, no adventitious roots were observed in either germplasm. However, numerous adventitious roots emerged from the submerged stem of MRS after prolonged waterlogging stress (about 6 weeks, data not shown).

### 2.2. The Effect of Waterlogging on the Photosynthesis

**Figure 2:**
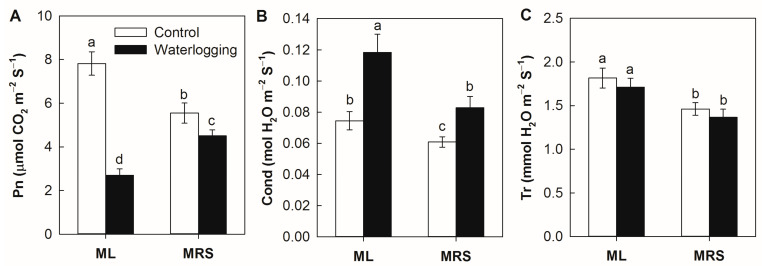
Gas exchange parameters of MRS and ML plantlets subjected to waterlogging stress for 3 weeks. (**A**) Photosynthetic rate, Pn; (**B**) Stomatal conductance, Cond; (**C**) Transpiration rate, Tr. MRS: Maorenshen, a wild germplasm from *Actinidia valvata* Dunn. ML: Miliang-1, a cultivar from *Actinidia chinensis* var. *deliciosa* (A.Chev.) A.Chev. Values are means ± SE (n = 4). Different letters indicate statistically significant differences at *p* < 0.05.

In the absence of stress, the photosynthetic rate (Pn), stomatal conductance (Cond) and transpiration rate (Tr) of the MRS plantlets were significantly lower than in the ML plantlets. The Pn decreased significantly under waterlogging stress in both germplasms, but the Pn was higher in MRS than in ML under waterlogging stress conditions (Figure 2A). Waterlogging stress induced significant increases in the Cond in both germplasms, but this change was much smaller in MRS than in ML (Figure 2B). Waterlogging stress had no effect on the Tr in both germplasms (Figure 2C).

### 2.3. The Effect of Waterlogging on the Chlorophyll Fluorescence Characteristics

**Figure 3:**
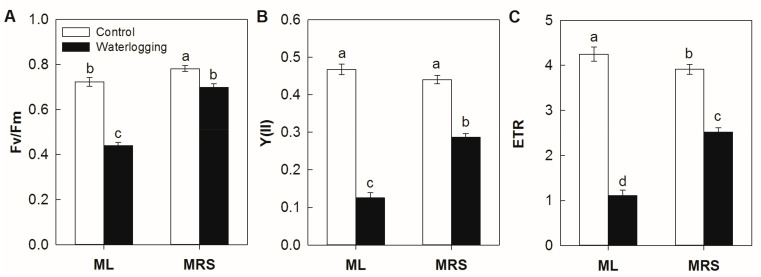
Chlorophyll fluorescence characteristics of MRS and ML plantlets subjected to waterlogging stress for 3 weeks. (**A**) Maximum efficiency of the PSII photochemistry, Fv/Fm; (**B**) Actual efficiency of PSII, Y(II); (**C**) Electron transfer efficiency of PSII, ETR. MRS: Maorenshen, a wild germplasm from *Actinidia valvata* Dunn; ML: Miliang-1, a cultivar from *Actinidia chinensis* var. *deliciosa* (A.Chev.) A.Chev. Values are means ± SE (n = 4). Different letters indicate statistically significant differences at *p* < 0.05.

No significant differences were observed among the germplasms under normal conditions. As shown in Figure 3, waterlogging stress consistently and significantly reduced the maximum efficiency of the PSII photochemistry (Fv/Fm) as well as the actual efficiency of PSII (Y(II)) and the electron transfer efficiency of PSII (ETR) in both germplasms, but the changes were less pronounced in the MRS plantlets than in the ML plantlets under waterlogging stress. The Fv/Fm, Y(II) and ETR of the MRS plantlets were 1.56-, 2.28- and 2.27-fold higher than those of the ML plantlets after 3 weeks of waterlogging stress.

### 2.4. The Effect of Waterlogging on the Root Activity

**Figure 4:**
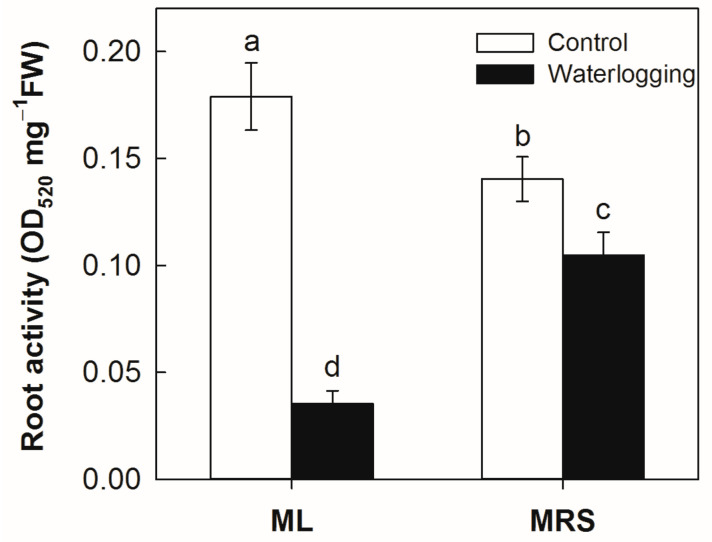
Root activity of MRS and ML plantlets subjected to waterlogging stress for 3 weeks. MRS: Maorenshen, a wild germplasm from *Actinidia valvata* Dunn. ML: Miliang-1, a cultivar from *Actinidia chinensis* var. *deliciosa* (A.Chev.) A.Chev. Values are means ± SE (n = 4). Different letters indicate statistically significant differences at *p* < 0.05.

Under normal conditions, the root activities of the MRS plantlets were significantly lower than that in ML plantlets (Figure 4). Waterlogging stress significantly reduced the root activities in both germplasms. However, the root activities were much higher in MRS than in ML under waterlogging stress. The root activity decreased by approximately 80.3% in the ML plantlets and by 25.3% in the MRS plantlets under waterlogging stress.

### 2.5. Anatomical Responses to Waterlogging

**Figure 5:**
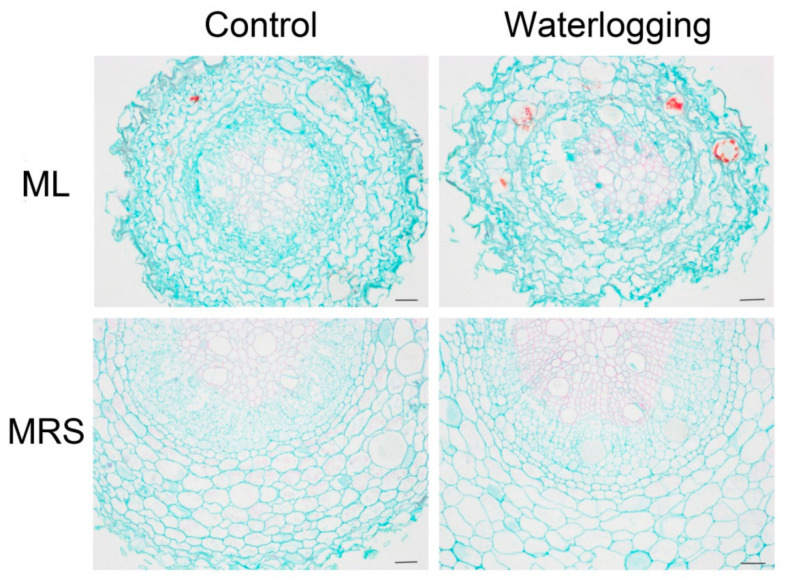
Transverse root anatomy of MRS and ML plantlets subjected to waterlogging stress for 3 weeks. MRS: Maorenshen, a wild germplasm from *Actinidia valvata* Dunn. ML: Miliang-1, a cultivar from *Actinidia chinensis* var. *deliciosa* (A.Chev.) A.Chev. Scale bars = 100 μm.

As shown in Figure 5, the root structure was similar and there was no aerenchyma in the root exodermis of the two kiwifruit germplasms. The only difference was in the cortical cell morphology under normal conditions. The root structure of MRS remained unchanged, whereas the majority of the ML roots rotted after 3 weeks of waterlogging. No aerenchyma appeared in either germplasm under waterlogging stress.

### 2.6. The Effect of Waterlogging on the PDC and ADH Enzyme Activities

**Figure 6:**
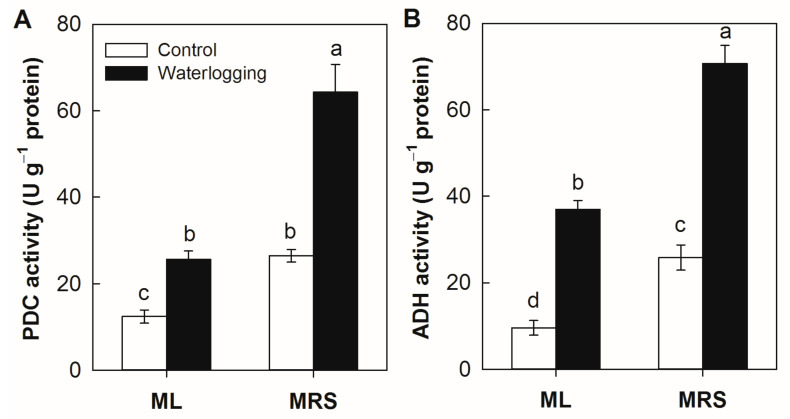
Enzyme activities of PDC (**A**) and ADH (**B**) in MRS and ML roots subjected to waterlogging stress for 3 weeks. PDC: pyruvate decarboxylase; ADH: alcohol dehydrogenase. MRS: Maorenshen, a wild germplasm from *Actinidia valvata* Dunn. ML: Miliang-1, a cultivar from *Actinidia chinensis* var. *deliciosa* (A.Chev.) A.Chev. Values are means ± SE (n = 4). Different letters indicate statistically significant differences at *p* < 0.05.

In the absence of stress, the PDC and ADH enzyme activities in the MRS roots were 113.5% and 150.4% higher than in the ML roots, respectively (Figure 6). Waterlogging stress significantly enhanced the PDC and ADH enzyme activities in both germplasms, but they were much higher in MRS than in ML under waterlogging stress.

### 2.7. The Effect of Waterlogging on the Expression of PDC and ADH

**Figure 7:**
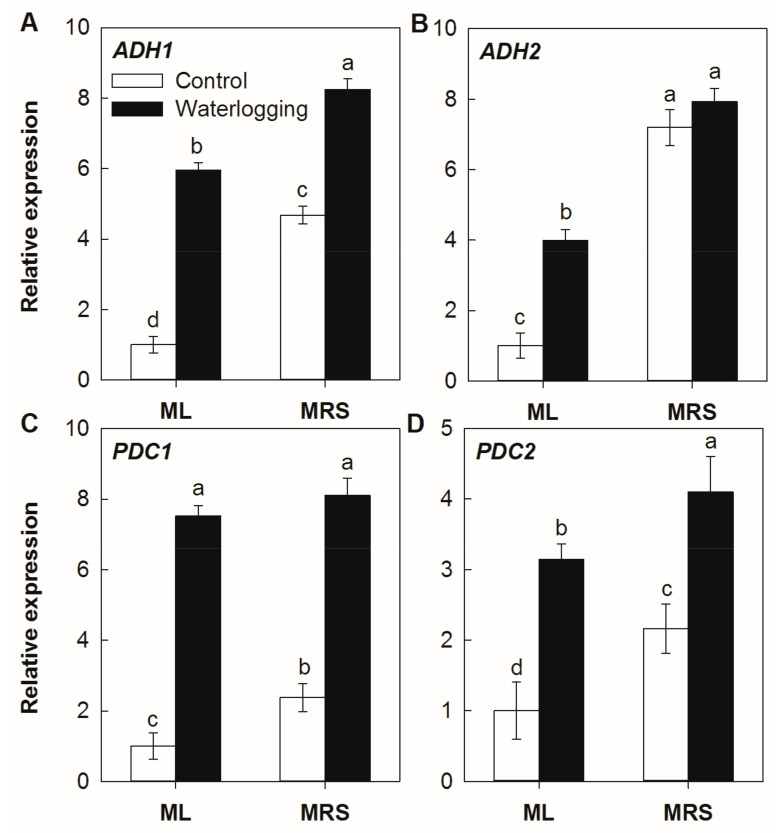
Expression levels of *PDC* and *ADH* genes in MRS and ML roots subjected to waterlogging stress for 3 weeks. (**A**) *Alcohol dehydrogenase 1*, *ADH1*; (**B**) *ADH2*; (**C**) *Pyruvate decarboxylase 1*, *PDC1*; (**D**) *PDC2*. MRS: Maorenshen, a wild germplasm from *Actinidia valvata* Dunn. ML: Miliang-1, a cultivar from *Actinidia chinensis* var. *deliciosa* (A.Chev.) A.Chev. Values are means ± SE (n = 3). Different letters indicate statistically significant differences at *p* < 0.05.

A qRT-PCR analysis showed that all four tested genes had a high constitutive expression in the MRS roots under normal conditions (Figure 7). In the absence of stress, the expression levels of *AcADH1*, *AcADH2*, *AcPDC1* and *AcPDC2* in the MRS roots were 3.68-, 6.20-, 1.37- and 1.16-fold higher than those in the ML roots, respectively. Waterlogging stress significantly upregulated the transcript levels of *AcPDC* and *AcADH* in both germplasms, except for *AcADH2* in the MRS roots. The transcript levels of *AcADH1*, *AcADH2*, *AcPDC1* and *AcPDC2* in the MRS roots were 38.5%, 98.6%, 7.7% and 30.4% higher than in the ML roots, respectively.

### 2.8. The Effect of Waterlogging on the H_2_O_2_ Accumulation and Antioxidant Abilities

**Figure 8:**
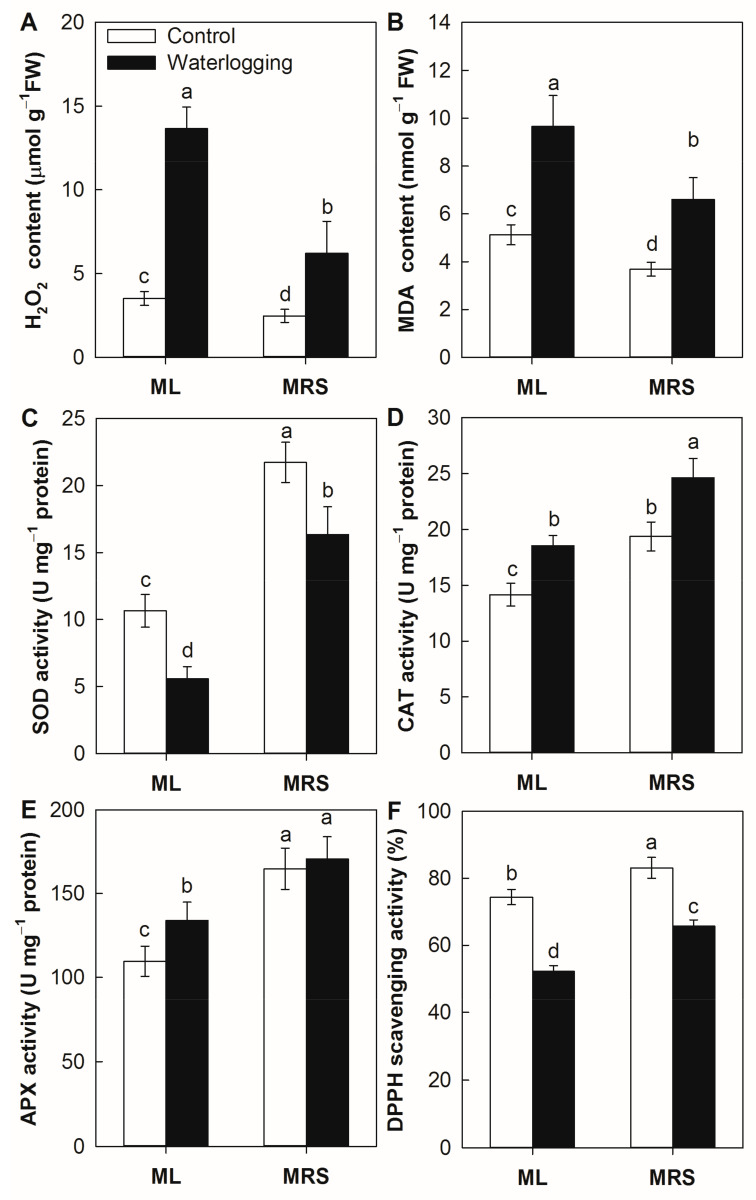
H_2_O_2_ accumulation and antioxidant abilities in MRS and ML roots subjected to waterlogging stress for 3 weeks. (**A**) H_2_O_2_ content; (**B**) MDA content; (**C**) Superoxide dismutase, SOD; (**D**) Catalase, CAT; (**E**) Ascorbate peroxidase, APX; (**F**) DPPH-radical scavenging activity. MRS: Maorenshen, a wild germplasm from *Actinidia valvata* Dunn. ML: Miliang-1, a cultivar from *Actinidia chinensis* var. *deliciosa* (A.Chev.) A.Chev. Values are means ± SE (n = 4). Different letters indicate statistically significant differences at *p* < 0.05.

As shown in Figure 8, waterlogging stress consistently and significantly induced the accumulation of H_2_O_2_ and MDA in both germplasms, but they were much lower in MRS than in ML under waterlogging stress. In the absence of stress, the superoxide dismutase (SOD), catalase (CAT) and ascorbate peroxidase (APX) enzyme activities were 104.0%, 36.8% and 50.4% higher in the MRS roots than in the ML roots, respectively. Waterlogging stress significantly reduced SOD enzyme activities but enhanced the CAT enzyme activities in both germplasms. APX enzyme activity was enhanced by waterlogging stress in ML roots, but remained unchanged in MRS roots. The SOD, CAT and APX enzyme activities were 193.1%, 32.8% and 27.4% higher in the MRS roots than in the ML roots under waterlogging stress, respectively (Figure 8C–E). Waterlogging stress significantly reduced DPPH-radical scavenging activities in both germplasms, but they were much higher in MRS than in ML under waterlogging stress.

## 3. Discussion

Waterlogging stress results in root hypoxia, which induces metabolites reduction, photosynthesis inhibition, anaerobic respiration and root injury or even plant death [6,10,11,22]. The rich genetic diversity and abundant wild germplasm resources among *Actinidia* plants are natural resources for improving the waterlogging tolerance of kiwifruit cultivars [20,21]. In the present study, the wild *A. valvata* germplasm MRS displayed fewer symptoms of an injury without obvious wilted leaves or rotten roots under waterlogging stress (Figure 1). The MRS plantlets showed higher photosynthetic rates, chlorophyll fluorescence characteristics and root activity under stress conditions (Figure 2, Figure 3 and Figure 4). These results indicate that MRS is tolerant to waterlogging and might be a promising genetic resource candidate for developing waterlogging-tolerant kiwifruit cultivars or rootstocks.

Waterlogging stress generally causes initial injuries to the root, which impact on the photosynthesis in the above-ground leaves and growth of the whole plant [6,8]. Roots are critical for absorbing and transporting water and nutrients. Root activity is an important indicator of plant waterlogging tolerance [11,23]. Reductions in root activity and the leaf photosynthetic rate are common responses of plants to waterlogging stress. Here, MRS plantlets displayed a higher root activity associated with better leaf performance under waterlogging stress (Figure 4). A high root activity during waterlogging contributes to the maintenance of the leaf photosynthesis, which in turn improves the root activity by supplying energy metabolism substrates. Thus, the maintenance of a high root activity might be responsible for the high waterlogging tolerance seen in the wild *A. valvata* germplasm MRS.

The formation of aerenchyma and the growth of adventitious roots are characterized as common anatomical responses in waterlogged plants [23,24,25,26,27]. These responses at an anatomical level facilitate oxygen capture for the submerged tissues, alleviate the hypoxic conditions and contribute to the survival of plants in frequently waterlogged soils [10,12,28,29]. In the present study, neither aerenchyma in the roots nor adventitious roots appeared in either germplasm after three weeks of waterlogging stress (Figure 1 and Figure 5). After prolonged waterlogging for about six weeks, numerous adventitious roots emerged from the submerged stem in MRS but not in the ML plantlets, which died (data not shown). These results indicate that the high tolerance to waterlogging of MRS was independent of the formation of aerenchyma and adventitious roots, especially in short- and medium-term waterlogging. The inability to form aerenchyma in kiwifruit plants might be one reason for their sensitivity to waterlogging.

Waterlogging affects numerous physiological and metabolic processes [13,22,30,31]. One of the primary pathways induced is anaerobic respiration for the production of ATP. Increases in alcohol fermentation catalyzed by PDC and ADH have been repeatedly observed in plants under waterlogging [1,10,13,14,15]. Ethanolic fermentation has been classically associated with flooding tolerance [4,6,10,15,23]. Recently, it was reported that two *PDC* and two *ADH* genes were induced in kiwifruit after a waterlogging treatment using Illumina sequencing technology, and the overexpression of either kiwifruit *ADH* or *PDC* from *A. deliciosa* could enhance the waterlogging tolerance in *Arabidopsis* [1,5,16,17]. Here, the activities of PDC and ADH as well as the expression levels of the corresponding genes were upregulated by waterlogging stress. The *PDC* and *ADH* genes were constitutively higher expressed in the wild *A. valvata* germplasm, MRS, together with high PDC and ADH enzyme activities under both normal and stress conditions (Figure 6 and Figure 7). These results suggest that the high constitutive and induced expression of *PDC* and *ADH* genes might be at least partly responsible for the high waterlogging tolerance of the wild *A. valvata* germplasm. The high constitutive activities as well as the induction of PDC and ADH might contribute to NAD^+^ regeneration and ATP supply under hypoxic stress, which helps to maintain root activity and plant growth.

Waterlogging stress triggers ROS accumulation and breaks down the balance between ROS generation and detoxification [11,32]. The accumulation of ROS can induce lipid peroxidation, chlorophyll degradation, and the loss of cell membrane integrity and photosynthetic activity [11]. Plants have developed an enzymatic antioxidant system (such as SOD, CAT, APX) and a nonenzymatic antioxidant system (such as ascorbate, flavonoids and secondary metabolites) to detoxify excess ROS and protect against ROS damage induced by environmental stress [32,33]. It has been identified that high antioxidant ability is critical for plant survival under waterlogging conditions [26]. In this study, the wild *A. valvata* germplasm MRS displayed higher activities of antioxidant enzymes, including SOD, CAT, and APX, as well as DPPH-radical scavenging ability, and decreased H_2_O_2_ and MDA accumulation under both normal and stress conditions (Figure 8). These results suggest that the high antioxidant ability might contribute to the high waterlogging tolerance of the wild *A. valvata* germplasm.

In summary, our results suggest that the metabolic adaptions with enhanced ethanolic fermentation and antioxidant abilities play an important role in the waterlogging adaption of the *A. valvata* germplasm. The constitutive and inductive activities of PDC and ADH, as well as antioxidant abilities might contribute to the growth and survival under waterlogging conditions. This study provides a better understanding of the physiological mechanisms involved in waterlogging tolerance in wild kiwifruit germplasm. However, the present results are derived from a preliminary comparative study, and further research to explore the key factors in waterlogging adaption and the underlying molecular mechanisms is needed. Moreover, further efforts to syncretize waterlogging tolerance from *A. valvata* germplasm into kiwifruit cultivars are promising and critical for kiwifruit breeding.

## 4. Materials and Methods

### 4.1. Material and Treatments

The kiwifruit germplasms were obtained from Germplasm Resources Nursery of Fujian Deciduous Fruits (Kiwifruit Nursery). Plantlets obtained from tissue culture with identical medium for two kiwifruit germplasms were planted in a plastic container (diameter × height: 110 × 140 mm) filled with autoclaved gardening soil and were cultured in a climate chamber at 25 °C with a 16/8 h light/dark cycle and 60% relative humidity. Three-month-old plants were used for the waterlogging treatments. The control group was cultured with normal irrigation. The treated groups were waterlogged with water 3–4 cm over the surface of the substrates and were kept under waterlogging stress for 3 weeks.

Twelve uniform plantlets for each germplasm per treatment were randomly divided into four biological replicates. All measurements were conducted after 3 weeks of waterlogging stress. The roots were immediately frozen in liquid nitrogen after carefully cleaning off the substrates and then stored at −80 °C for further use.

### 4.2. Gas Exchange Parameter Analysis

The most recent fully developed leaves (approximately the third leaf from the top) were selected to measure the gas exchange parameters using a portable photosynthesis system (Li-6400; LI-COR Inc., Lincoln, NE, USA) according to Chen et al. [34]. The leaf was placed in a 6 cm^2^ chamber at a photo flux density of 300 μmol m^−2^s^−1^. Four plantlets of each germplasm per treatment were analyzed.

### 4.3. Chlorophyll Fluorescence Analysis

The chlorophyll fluorescence characteristics of individual leaves were analyzed using a pulse amplitude-modulated chlorophyll fluorescence system (Imaging PAM, Walz, Effeltrich, Germany) according to Chen et al. [34]. The most recent fully developed leaves were dark-adapted for 30 min before the measurement. Four plantlets of each germplasm per treatment were analyzed.

### 4.4. Root Activity Analysis

The root activity was determined using a 2,3,5-triphenyltetrazolium chloride (TTC) reduction assay according to Yamauchi et al. [35]. Fresh root segments were incubated in a 0.6% (*w*/*v*) TTC solution at 40 °C for 30 min and then rinsed with deionized water. The root segments were incubated overnight at room temperature in 95% (*v*/*v*) ethanol. The reduction of TTC was expressed as the absorbance of the extracted solutions at 520 nm in a spectrophotometer (UV-2550, Shimadzu, Kyoto, Japan).

### 4.5. Root Anatomical Analysis

Root segments (3.0–4.0 cm from the root tip) were fixed in formalin/alcohol/acetic acid (FAA) for 24 h. The material was then dehydrated by being passed through an ethanol series, cleaned in xylene and embedded in paraffin wax. Transverse 8 μm-thick sections were obtained using a rotary microtome (LEICA RM2016, Wetzlar, Germany) then stained with safranine and fast green and photographed with a Nikon SMZ18 microscope (Nikon, Tokyo, Japan).

### 4.6. ADH and PDC Activities Analysis

Frozen root sections (3.0–5.0 cm long) were ground to a powder and stored at −70 °C until use. The extraction of the materials and the measurement of activities of the ADH and PDC were performed according to Yin et al. [36]. A sum of 1 U of ADH and PDC was defined as the amount of enzyme required to decompose 1 μmol of NADH per g/tissue per minute.

### 4.7. Expression Analysis

Frozen root samples were used to measure the transcription levels of *AcADH* and *AcPDC*, which were identified from the Kiwifruit Genome Database. The RNA was isolated and the gene expression was analyzed using a quantitative RT-PCR as described by Chen et al. [37], using *AcActin* and *AcEF1α* as internal references. The amplification efficiency of the primers was determined by a standard curve 10 times the gradient dilution of the cDNA template from the ML root samples under normal conditions. The specificities of these primers were verified via melt curve analysis. The relative expression was calculated using the 2^−ΔΔCt^ method. As similar results were obtained using both reference genes, only the results based on *AcActin* were presented. There were three replicates in each treatment. The genes and the sequences of their specific primers are listed in Table 1.

### 4.8. Hydrogen Peroxide (H_2_O_2_) and Lipid Peroxidation Analysis

Frozen materials (~200 mg) were homogenized in 5 mL of 0.1% (*w*/*v*) trichloroacetic acid (TCA, AR ≥ 99.0%). The homogenate was centrifuged at 12,000× *g* for 15 min at 4 °C. For the determination of H_2_O_2_, the supernatant (0.5 mL) was added to 0.5 mL of 10 mM potassium phosphate buffer (pH 7.0) and 1 mL 1 M KI (AR ≥ 99.5%). The absorbance of supernatant was measured at 390 nm. The content of H_2_O_2_ was calculated using a standard curve as described by Velikova et al. [38]. For the determination of MDA, the supernatant (0.5 mL) was mixed with 0.5 mL of 0.6% thiobarbituric acid (TBA, BR ≥ 98.5%) and incubated for 15 min in boiling water. Then, the mixture was cooled on ice rapidly and centrifuged again. The absorbance of supernatant was measured at 450 nm, 532 nm and 600 nm, and the MDA content was determined via the TBA reaction according to Draper et al. [39].

### 4.9. Antioxidant Abilities Analysis

Frozen samples (~200 mg) were homogenized with 2.5 mL of 50 mM sodium phosphate buffer (pH 7.2). The homogenate was centrifuged at 12,000× *g* for 15 min at 4 °C and the supernatant was used for determination the activity of antioxidant enzymes [40]. Superoxide dismutase (SOD) activity was estimated based on its ability to inhibit the photochemical reduction of nitro blue tetrazolium (NBT, BR ≥ 98.0%) at 560 nm [41]. Catalase (CAT) activity was estimated by measuring the initial rate of the disappearance of H_2_O_2_ (GR ≥ 30.0%) at 240 nm [38]. Ascorbate peroxidase (APX) activity was estimated based on the decrease in absorbance at 290 nm as the ascorbate (AR ≥ 99.5%) is oxidized [42]. DPPH-radical scavenging activity was measured and calculated according to Matsuura et al. [43].

### 4.10. Statistical Analysis

The statistical analysis was performed using the SPSS statistics software (Version 19.0 for Windows, SPSS, Chicago, IL, USA). The data were analyzed via analysis of variance (ANOVA) using the least significant differences (LSD) post hoc test, and a *p* < 0.05 was considered to be significant.

## Figures and Tables

**Table 1:** Genes and oligonucleotides used in the real-time quantitative PCR experiment.

Gene	Gene ID	Primer
*AcADH1*	*Acc08063*	F:5′-CCATCCGATGAATCTCTTGAA-3′ R:5′-CGAAAGCCTTGTTGATCTCAG-3′
*AcADH2*	*Acc11566*	F:5′-CAAGACACACCCGATGAATTT-3′ R:5′-CCGAGAACGAGACTTGATGAG-3′
*AcPDC1*	*Acc01807*	F:5′-GAAGCAATTGACACAGCAACA-3′ R:5′-CCGTACACACAAGCAGCAGTA-3
*AcPDC2*	*Acc17284*	F:5′-GAGCAAAGAGCTGCTGGAAT-3′ R:5′-TACATCCCATTGACCAGGAAA-3′
*AcActin*	*Acc08081*	F:5′-TGCATGAGCGATCAAGTTTCAAG-3′ R:5′-TGTCCCATGTCTGGTTGATGACT-3′

## Data Availability

Not applicable.
